# Whole-genome resequencing of major populations revealed domestication-related genes in yaks

**DOI:** 10.1186/s12864-024-09993-7

**Published:** 2024-01-17

**Authors:** Wei Peng, Changqi Fu, Shi Shu, Guowen Wang, Hui Wang, Binglin Yue, Ming Zhang, Xinrui Liu, Yaxin Liu, Jun Zhang, Jincheng Zhong, Jiabo Wang

**Affiliations:** 1 Qinghai Academy of Animal Science and Veterinary Medicine, Qinghai University, Xining, 810016 China; 2 Key Laboratory of Qinghai-Tibetan Plateau Animal Genetic Resource Reservation and Utilization (Sichuan Province and Ministry of Education), Southwest Minzu University, Chengdu, 610041 China

**Keywords:** Yaks, Population stratification, Selective sweep, Domestication

## Abstract

**Background:**

The yak is a symbol of the Qinghai-Tibet Plateau and provides important basic resources for human life on the plateau. Domestic yaks have been subjected to strong artificial selection and environmental pressures over the long-term. Understanding the molecular mechanisms of phenotypic differences in yak populations can reveal key functional genes involved in the domestication process and improve genetic breeding.

**Material and method:**

Here, we re-sequenced 80 yaks (Maiwa, Yushu, and Huanhu populations) to identify single-nucleotide polymorphisms (SNPs) as genetic variants. After filtering and quality control, remaining SNPs were kept to identify the genome-wide regions of selective sweeps associated with domestic traits. The four methods (π, XPEHH, iHS, and XP-nSL) were used to detect the population genetic separation.

**Results:**

By comparing the differences in the population stratification, linkage disequilibrium decay rate, and characteristic selective sweep signals, we identified 203 putative selective regions of domestic traits, 45 of which were mapped to 27 known genes. They were clustered into 4 major GO biological process terms. All known genes were associated with seven major domestication traits, such as dwarfism (*ANKRD28*), milk (*HECW1*, *HECW2*, and *OSBPL2*), meat (*SPATA5* and *GRHL2*), fertility (*BTBD11* and *ARFIP1*), adaptation (*NCKAP5*, *ANTXR1*, *LAMA5*, *OSBPL2*, *AOC2*, and *RYR2*), growth (*GRHL2*, *GRID2*, *SMARCAL1*, and *EPHB2*), and the immune system (*INPP5D* and *ADCYAP1R1*).

**Conclusions:**

We provided there is an obvious genetic different among domestic progress in these three yak populations. Our findings improve the understanding of the major genetic switches and domestic processes among yak populations.

**Supplementary Information:**

The online version contains supplementary material available at 10.1186/s12864-024-09993-7.

## Background

The yak (*Bos grunniens*) is a unique symbol of the Qinghai-Tibet Plateau [1]. The more than 15 million domestic yaks living in this area provide basic resources for the nearly 10 million humans in Tibet and Qinghai provinces and near Sichuan province. Yaks were domesticated from wild yaks by ancient nomadic people, possibly beginning in the time of the Longshan Culture during the late New Stone Age [2]. Currently, a major population of domestic yaks inhabits the Hengduan Mountain between Sichuan and Tibet provinces and around Kunlun Mountain between Qinghai and Tibet provinces. Among these domestic populations, Maiwa, Yushu, and Huanhu yaks are major distribution populations, and exhibit differences in their body development, habitat adaptation, economic production, and other traits [3, 4]. The hybridization is the most effective approach to improve production or change physical state. However, the male offspring produced by mating between yaks and cattle are sterile, making it difficult to identify functional genes and improve hybridization. A feasible way is based on the deep sequencing technology to compare yak population separation to reveal underlying key functional genes.

Following the development and expansion of high-throughput sequencing technology, the yak reference genome was published in 2012 [4]. Hence, the biological markers, such as single-nucleotide polymorphisms (SNPs), are always used to descript difference and separation between species or populations. Genomic comparisons between populations of closely related species have provided insights into the genetic basis and selective sweep under environmental pressure and artificial selection [1]. In 2015, Qiu et al. revealed a population selection sweep by comparing wild and domestic yaks [5]. However, because of there was not complete whole reference genome at the chromosome level, these identified genes were not revealed clearly. Recently, the reference genome at the chromosome level has been published [6, 7]. The reference genome at the chromosome level could bring more important genome analysis for yaks genetics, such as linkage region with continuous physical location information, linkage disequilibrium (LD) decay rate, and selective sweep signals. Though there is rarely research about yaks population selective sweep signals, many candidate genes were identified in domestic cattle. For example, Taye et al. [8] reported candidate genes associated with heat tolerance between closer sib of African cattle populations. R.Li et al. [9] identified selective signature in Dehong humped cattle for heat tolerance.

In this study, we re-sequenced and obtained the whole genomes variants of 80 yaks (from the Maiwa, Yushu, and Huanhu yak populations). Following the population genetic research approach, we determined their genomic variability, population stratification, LD decay rate, and characteristic selective sweep signals of past domestic processes. Our main objective was to identify candidate functional genes that may contribute to economic traits, adaptation to living in the plateau, and the response to artificial selection of shepherds in the Qinghai-Tibet Plateau. We report a detailed selective sweep signature between the whole-genome and provide insight into the evolutionary history of these yaks.

## Material and methods

### Sample collection

**Fig. 1:**
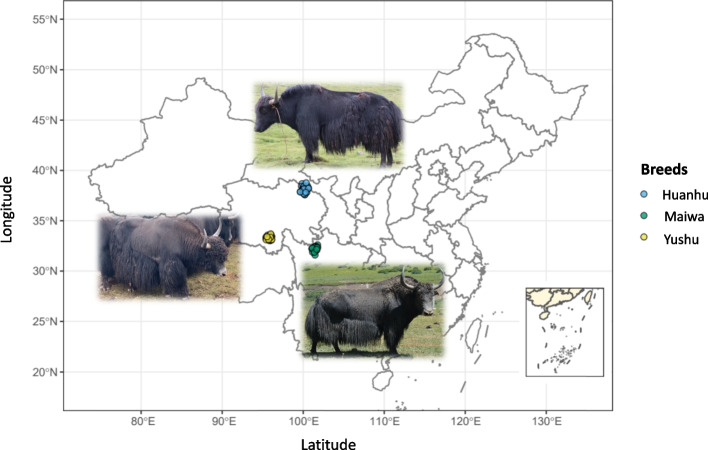
The geographic location and posture graph of samples in three yak populations. All 80 male yaks were sampled from Gangcha city (Huanhu yaks), Hongyuan city (Maiwa yaks), and Yushu city (Yushu yaks) in China. These three populations are the most widely distributed populations in the Northeast and East of Qinghai-Tibet Plateau

To generate a yak population for selective sweep detection, we collected individuals from Yushu city in Qinghai 2021 (altitude: 4500 m, *n* = 25), Gangcha city in Qinghai 2021 (altitude: 3300 m, *n* = 30), and Hongyuan city in Sichuan 2020 (altitude: 3500 m, *n* = 25) (Fig. 1). All yaks were all male adults (Fig. 1) of similar age (4–5 years). The three yak populations within the five generations were genetically unrelated and are the three major domestic populations distributed on the Tibetan Plateau. They are also the main representatives of adaptation to each altitude and environment. Ear tissues (approximately 1 cm^3^) were sampled and stored in 75% medicinal alcohol for DNA extraction.

### Genome sequencing and single-nucleotide polymorphism calling

After restriction enzyme digestion to break whole genome into reads, at least 3 µg DNA was linked with barcode adapters to construct a sequencing library according to the manufacturer’s instructions (Illumina, San Diego, CA, USA). An Illumina NovaSeq 6000 was used to sequence the libraries at Compass Agritechnology Co., Ltd. (Beijing, China), and 150 bp paired-end reads were generated with an insert size of approximately 350 bp. A dataset of 1.2 Tb was obtained through pair-end sequencing. The raw data were quality-filtered using fastp software (version 0.19.8) with default parameters [10]. Clean sequencing reads were mapped to the yak reference genome (BosGru 3.0) [7] using the BWA-MEM function (version 0.7.17). SAMtools software was used to convert the SAM format files generated by BWA into BAM format and to sort and mark duplicate reads. The Genome Analysis Toolkit was used to identify genotype variations containing SNPs and insertions-deletions. In addition, potential polymerase chain reaction duplications were removed using the SAMtools command “rmdup”. If multiple read pairs contained identical external coordinates, only the pair with the highest mapping quality was retained. The VCF files from each individuals, including all genetic variates, were merged into a compilation file using the Genome Analysis Toolkit (GATK) [11]. In this compilation VCF file, markers with a missing rate of > 10% were excluded through filtering. All remaining markers (missing rate < 10%) were used to impute the missing genotype values using Beagle software (version 1.3.2) with default parameters [12].

### Genotype view and population stratification

After quality control, the VCF file was converted to HapMap format files using a Perl code written in our laboratory (on the GitHub website https://github.com/jiabowang/Converting). Only ﻿SNPs were used to analyze the genotype distributions and perform population stratification, the other variation types markers were removed. The distance and genotype correlation between neighboring SNPs were used to determine the degree of coverage of the sequences. The whole-genotype view and three dimensionality principal component analysis (PCA) were performed using GAPIT software [13]. In the GAPIT, all distance and correlation values were filtered by outlier remove function, because the values of markers among previous and next chromosomes should be weeded. LD decay was calculated using PopLDdecay software [14]. Each of the 300 SNPs was considered as a window to calculate the average correlation square values. Following the window, the whole genome was scanned from the first SNP to the final SNP. In the final calculation results, the number of values should be equal to the number of total SNPs minus 300. The final distance and correlation values are the average values in each 300 SNPs window.

### Genome-wide selection sweep

To identify the genome-wide regions of selective sweeps associated with domestic traits, we used several methods to investigate the selection signatures, including examining the nucleotide diversity (π), cross-population extended haplotype homozygosity (XPEHH), integrated haplotype score (iHS), and cross-population haplotype-based (XP-nSL). These four analyses were performed using Selscan software. The VCF file containing all individual genotype variants was divided into three sub-files for each population. The BCFtools, VCFtools, and Beagle tools were used to convert the data format and to perform phasing. A sliding window size of 100 bp was used to calculate the π, XPEHH, iHS, and XP-nSL values. The gap between 0 and these values can be used to distinguish the level of separation. This separation is a relative level. For the calculation of π and iHS values, the genome variants within whole population were used, and the separation levels of π and iHS values could be considered as signals of “within” type. For the calculation of XPEHH and XP-nSL values, the genome variants between three populations were used, and the separation levels of XPEHH and XP-nSL values could be considered as signals of “between” type. The pairwise of these three populations were used to calculate “between” separation levels. 

In this study, the maximum offset values in each pairwise comparison was considered as estimated selective sweep values in the whole population. To clearly show the results and reduce the figure size, the whole-genome was divided into bins. Each bin contained a 100 kb fragment. The maximum absolute values in the bin values were used to represent the bin values. Based on this “bins” strategy, the values can be compared between these four methods. Values 30,000 away from zero were retained to draw a figure. A value of 0.1% of the whole value was used as the cutoff threshold for significance.

### Functional gene annotation

Protein-coding genes in these outlier points were annotated using the yak reference genome (BosGru 3.0). All gene names within 100 kb regions were treated as candidate positively selected genes. Using Metascape software, we performed Gene Ontology (GO) pathway analysis of the positively selected genes to evaluate their biological functions.

## Results

### Sequencing and genotype statistics

**Fig. 2:**
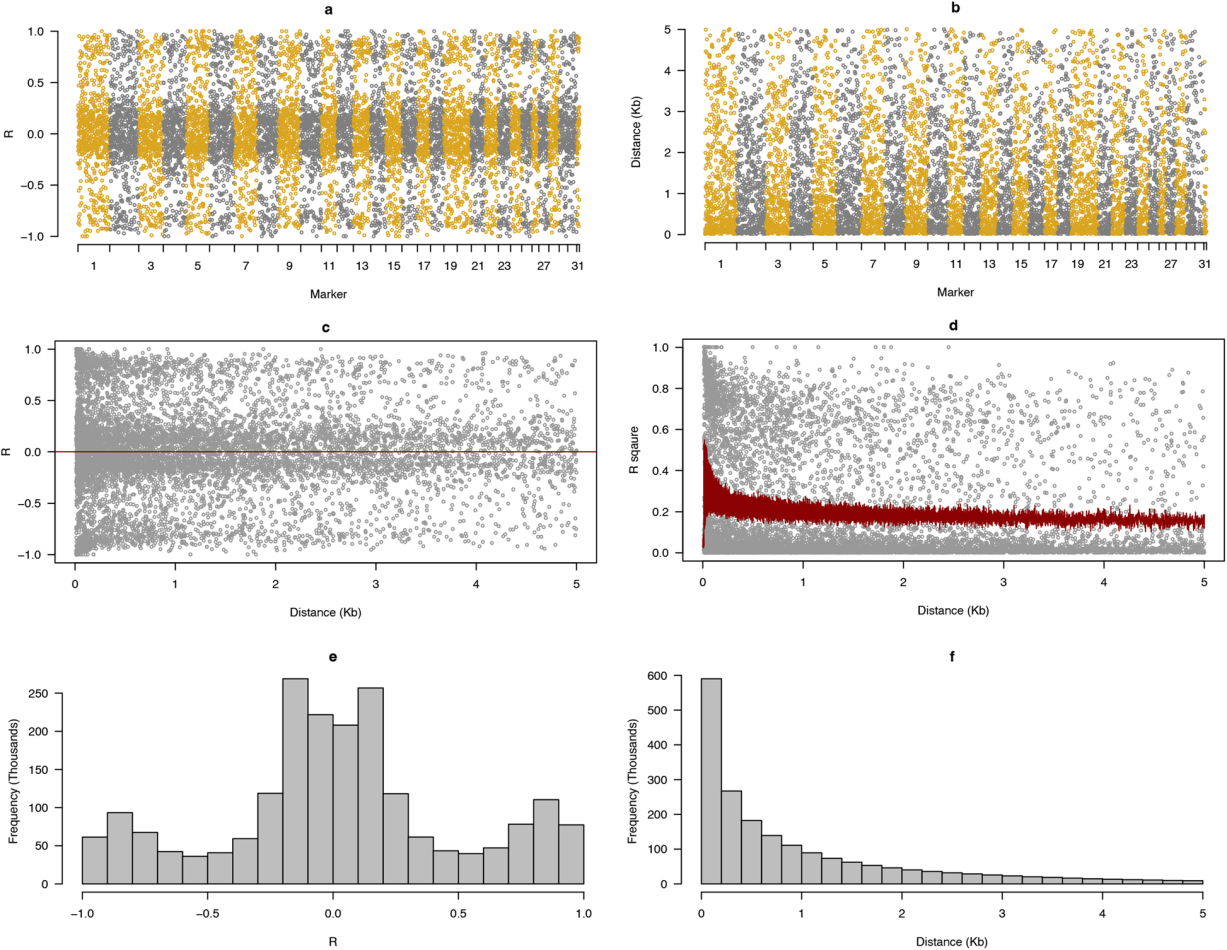
The genotype analysis view of 2 M markers in the whole genome sequencing. After sequencing and filtering, total 2,060,483 markers were kept in the whole genome. 29 autosomes and 2 sex chromosome were marked as numeric 1 to 31. The odd number of chromosome were painted as goldenrod color. The even number of chromosome were painted as gray color. The genotype and position of neighboring two markers were used to calculate correlation (R, **a**) and distance (**b**). The relative distance against R (**c**) and R^2^ (**d**) between markers were used to show the linkage level. Every 20 markers were used to calculate average values of R^2^, then the average R^2^ were used to indicate LD-decay of whole population. The frequency of R (**e**) and distance (**f**) were used to show the statistical results

We obtained 1.2 Tb sequencing data. The resequencing data have been deposited to the NCBI BioProject under accession no. is PRJNA899924. The resulting coverage per genome was 13 to 17 × , with a mean coverage of 15 × . After quality control and data filtering (removing markers with a missing rate > 0.05, minor allele frequency < 0.05), 2,060,483 SNPs were identified from all variants in the whole-genome resequencing data, which were distributed on 29 autosomes and 2 sex chromosomes (Fig. 2a, b). We coded these number of chromosomes as 1 to 31. Nearer markers were more correlated with each other compared to distant markers (Fig. 2c). The LD rapidly decreased to *R*^2^ < 0.2 within 0.83 kb (Fig. 2d), which is comparable to values reported in other populations. The frequency of the correlation and distance between each marker showed a statistical distribution (Fig. 2e, f). Comparison of allele frequency among these three populations indicated similar distribution (Fig. S1). The kinship of whole population based on the SNPs also did not show population separation (Fig. S2).

### Population stratification

**Fig. 3:**
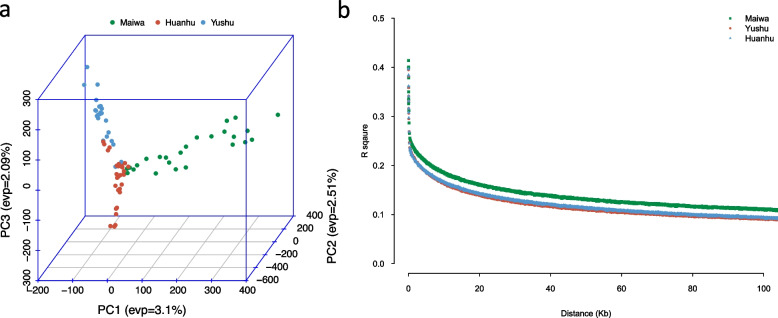
The 3 dimensionality PCA and LD decay of three yaks population. The whole genome markers were used to calculate principle component analysis (PCA). The top 3 PCs were used to draw the population structure (**a**). The red, blue, and green dots indicate Huanhu, Yushu, and Maiwa yaks population respectively. The R^2^ was used to descript the different of LD decay speed (**b**)

Most of yaks belong to three major clustering population developed from the 2,060,483 SNPs (Fig. S3). The first PC can explain 3.2% of the total variation, followed by 2.5% and 2.2% for the second and third PCs, respectively (Fig. S4). The lower ratio values of explained total variation by top three PCs indicated that there is separation inside of each population. Although, two-dimensional PCs plot could not distinguish Huanhu and Yushu yaks population, a three-dimensional PCs plot could be used to show the clustering results for all yak individuals (Fig. 3a). That phenomenon may be caused by much closer genetic relationship between Huanhu and Yushu yaks than Maiwa.

The similar results are appearing in the LD decay results. The results of LD decay analysis also demonstrated that this population was separated (Fig. 3b). The collection location of the Maiwa yaks was Hongyuan city, which is distant from the other two populations (Yushu and Huanhu). The LD decay speed of the Maiwa yak genome was lowest among the three populations. Based on these observations, certain genomic regions encoding domestic and adaptive traits may explain the variation in the population. Based on the Fig. 3a and b, these three yaks populations can be observed separation. However, the LD decay of Yushu and Huanhu yaks populations are similar, the separation of them can be observed in the 3d-PCs plot (Fig. 3a). That indicates there is obvious separation between these three population, or we can say they are separating.

### Selective sweep

**Fig. 4:**
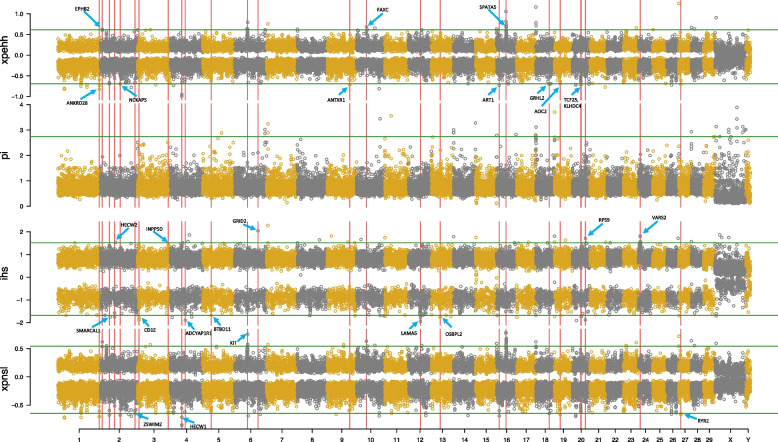
Genome-wide selective sweep and functional annotations. The odd number of chromosome were painted as goldenrod color. The even number of chromosome were painted as gray color. 0.1% of each whole values was used to filter significant selective sweeps (green line). Red vertical lines indicate functional genes above or below threshold cutoff

A total of 28,028 common positions was retained among the four methods. Windows with significantly high values (top 0.1%, XPEHH > 0.6131, Pi > 2.7447, iHS > 1.5197, XP-nSL > 0.5441) and low values (tail 0.1%, XPEHH < -0.6938, iHS < -1.6801, XP-nSL < -0.6447) were considered as the target windows in domestic yaks (Fig. 4). By applying these standards, we identified 203 regions on autosomes with high elevations in these four values (Table S1). After taking the intersection in a window-size (100 kb) between these four parameters and merging neighboring windows into the selected regions, 155 putative selective regions were identified in the whole genome.

### GO with signals

**Fig. 5:**
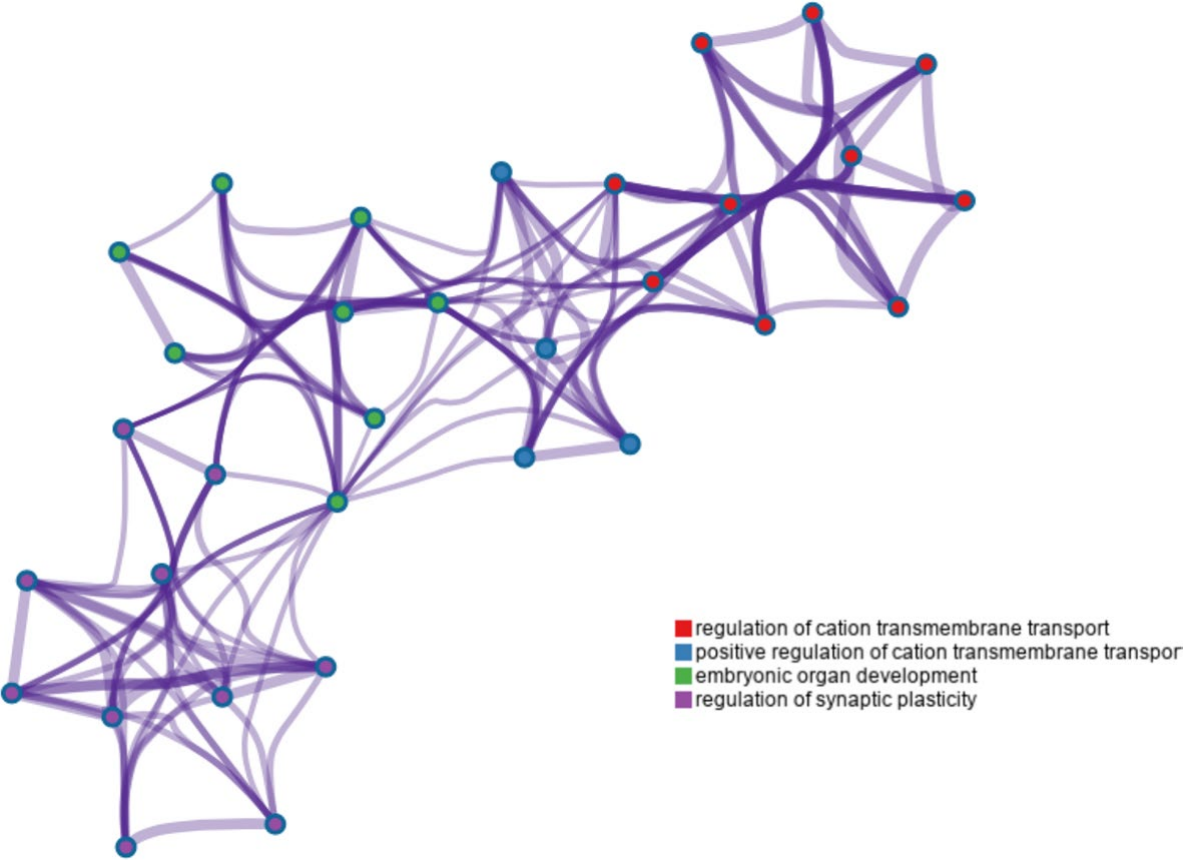
Enriched network between Gene Ontology terms. Total four major term type shows significant enrichment. A subset of enriched terms have been selected and rendered as a network, where sub-terms with a similarity more than 0.3 are connected by edges

Among these putative selective regions, 45 were mapped to 27 known genes in the yak reference genome (Table S2), which are marked with red lines in Fig. 4. The genes were named according to the detected signals. The genes were clustered into four GO biological process terms (regulation of cation transmembrane transport, positive regulation of cation transmembrane transport, embryonic organ development, and regulation of synaptic plasticity). We selected a subset of representative terms from the full cluster and converted the terms into a network layout (Fig. 5). Specifically, each term is represented by a circle node, where its size is proportional to the number of input genes falling under that term; its color represents its cluster identity (i.e., nodes of the same color belong to the same cluster). Terms with a similarity score > 0.3 are linked by an edge (thickness of the edge represents the similarity score). The network was visualized in Cytoscape with “force-directed” layout and with edge bundling for clarity. All enriched GO terms were significant (*p* < 0.01), as shown (Fig. S5 and S6).

## Discussion

All samples in this study were selected from central production areas in Hongyuan, Yushu, and Gangcha. Their genetic background was clear, and the yaks were genetically unrelated within five generations. Based on the 2-d PCA plot, comparison of MAF distribution, and kinship clustering, these three yaks population were not separated clearly. However, when the 3rd PC was added into 3-d PC plot, the 3 populations were divided. From the 3D PCA figure (Fig. 3A), these three populations share a region in the central of 3D space, and obvious separation in other three direction. That means they may come from some population and are developing to different, or they may come from different population and are developing to similar. Based on the previous study of that all yaks breeds could come from one original ancient population, we think that should be the former opinion. The more separations in Maiwa yaks means more mutations. The most of distances between the sweeps signals type in the “between” and “within” are more than 50 Kb, that means there is rarely regions could play both effects in the “between” and “within”. That phenomenon indicates these three populations are separating, and the separation factor is growing. The most of separation should be in some representation, behavior, or conduction.

Their genome average LD decay speed and PCA clustering results were similar to those of their geographical habitat. The Yushu and Huanhu yak populations were close to each other and exchanged genes much more frequently, whereas the Maiwa yak was far from the previous two populations. Genetic diversity is an integral factor influencing biological development and evolution [8, 9]. Selective sweep signals can indicate functional regions under environmental pressure and artificial selection [10–12]. These three populations also showed differences in their morphological, physiological, and other economic traits such as body size, weight, and hair in adults [13, 14].

A major goal of this study was to detect candidate genes affecting domestication traits in yak populations. All candidate genes were associated with seven major domestication traits, including dwarfism (*ANKRD28*), milk (*HECW1*, *HECW2*, and *OSBPL2*), meat (*SPATA5* and *GRHL2*), fertility (*BTBD11* and *ARFIP1*), adaptation (*NCKAP5*, *ANTXR1*, *LAMA5*, *OSBPL2*, *AOC2*, and *RYR2*), growth (*GRHL2*, *GRID2*, *SMARCAL1*, and *EPHB2*), and immunity (*INPP5D* and *ADCYAP1R1*). Other genes without previously relationships could not be identified for their role in domestication. The major reason of this phenomenon may be that the yak reference genome has not been perfectly annotated. Many regions in the genome were unknown gene or unknown functional. Based on our discovery, these region without known genes also are strong selection sweeps, although we have no idea about their functional and structural conclusion. Therefore, more extensive and in-depth research is needed.

Specific genetic diseases and milk production traits may be important factors in yak domestication. *ANKRD28* (ankyrin repeat domain 28) is related to pathways such as vesicle-mediated transport and transport to the Golgi and subsequent modification. ﻿According to the Genome Aggregation Database (https://gnomad.broadinstitute.org/), *ANKRD28* belongs to the class of loss-of-function haploinsufficiency genes, resulting in dwarfism in Holstein calves [15]. ﻿*HECW1* and *HECW2* are derived from the HECT, C2, and WW domains containing the E3 ubiquitin protein ligase gene family. In genetic research of cattle, *HECW1* was reported to be related to genetic substitution between Hanwoo and Holstein cows [16]. In 2017, *HECW2* was shown to be related to milking speed in French Holstein cows [17]. *OSBPL2* (oxysterol-binding protein like 2) is strongly associated with the lactose content and freezing point in Russian Holstein cattle according to GWAS results [18]. This gene and *LAMA5* (laminin subunit alpha 5) can also increase lactation persistence in the mammary gland [19]. *GRID2* (glutamate ionotropic receptor delta-type subunit 2) is associated with the central suspensory ligament in Chinese Holstein cows [20]. The central suspensory ligament is a major index of udder conformation and milking levels.

Meat production, body growth development, and fertility traits are major concerns in the Maiwa, Yushu, and Huanhu yak breeding programs. *SPATA5* (spermatogenesis associated 5) is associated with catalytic activity in Swiss [21] and meat traits in Hu sheep [22]. *GRHL2* (grainyhead-like transcription factor 2) has a maternal-related effect on the intramuscular fat content in cattle [23]. Additionally, gene regulation is involved in human and mouse embryo development [24]. In contrast to in previous studies, *GRID2* was strongly associated with dry matter intake, average daily gain, birth weight, and milk fat yield in cattle [25–28]. *SMARCAL1* (SWI/SNF related, matrix-associated, actin-dependent regulator of chromatin, subfamily a-like 1) is an *SNF2* family member and can increase 8-cell development in human, mice, and cattle embryos [29]. Zhe et al. identified *EPHB2* (EPH receptor B2) as a candidate gene associated with litter weight during weaning in Duroc pigs [30]. Based on a large-scale GWAS using 294,079 first-lactation Holstein cows, *BTBD11* (BTB domain-containing 11) was identified as a candidate gene with a major additive effect on the daughter pregnancy rate [31]. Shi-Yi et al. found that *ARFIP1* (ADP ribosylation factor-interacting protein 1) is associated with fertility and reproduction traits using whole-genome sequence genotypes [32].

In our three yak populations, altitude and environmental adaptation may have also participated in yak domestication as natural factors. *INPP5D* belongs to the inositol polyphosphate-5-phosphatase family and is involved in microglial activation, neuroinflammation, and immune responses in cattle [33, 34]. *ADCYAP1R1* (ADCYAP receptor type I) is a candidate gene for the immune response system in cattle [35]. The flight speed test is among the most commonly used behavioral methods to assess cattle temperament to reflect aspects of the environment. A large GWAS of 162,645 Nellore cattle performed by Silva Valente et al. showed that *NCKAP5* (NCK-associated protein 5) was associated with flight speed test scores. *ANTXR1* (anthrax toxin receptor 1) is the receptor for SVA in human and pig cells [36]. *AOC2* (amine oxidase copper containing 2) is a candidate gene associated with feed efficiency according to a GWAS in cattle [37]. Ca^2+^-release channels may be regulated by the expression level of *RYR2* (ryanodine receptor 2) and are associated with high-altitude adaptation in animals [38].

## Conclusion

There is the sufficient evidence for that the Maiwa, Huanhu, and Yushu yaks populations are separating from each other. Maiwa yaks are the most affected by domestication and selection press. The average LD distance was estimated as 0.83 kb. 45 of selective sweeps were annotated to 27 functional genes associated with domestication traits, such as dwarfism (*ANKRD28*), milk (*HECW1*, *HECW2*, and *OSBPL2*), meat (*SPATA5* and *GRHL2*), fertility (*BTBD11* and *ARFIP1*), adaptation (*NCKAP5*, *ANTXR1*, *LAMA5*, *OSBPL2*, *AOC2*, and *RYR2*), growth (*GRHL2*, *GRID2*, *SMARCAL1*, and *EPHB2*), and the immune system (*INPP5D* and *ADCYAP1R1*). These results improve the understanding of the evolutionary history of domestic yaks and provide valuable resources for genetic switches and breeding progress.

### Supplementary Information

**Additional file 1: Figure S1.** MAF distribution of Maiwa, Yushu, and Huanhu yaks. **Figure S2.** The kinship of whole population based on the SNPs. **Figure S3.** 2 D PCA plot for three yaks population. The A was drawn with PC1 against PC2. The B was drawn with PC1 against PC3. The C was drawn with PC3 against PC2. **Figure S4.** The percentage between PCs explained variance and total phenotype variance. **Figure S5.** Enriched network between Gene Ontology terms by *P*-values. **Figure S6.** Gene Ontology terms by *P*-values. Then 0.3 kappa score was applied as the threshold to cast the tree into term clusters. **Figure S7.** The LD decay of three yaks population with adjust R2 values. **Table S1.** Detected sweeps and associated gene names. **Table S2.** GO terms and descriptions.

## Data Availability

The resequencing data have been updated to NCBI BioProject accession NO. is PRJNA899924.
