# Supplementary material for: Whole-genome resequencing of major populations revealed domestication-related genes in yaks
Source: BMC Genomics. 2024 Jan 17;25:69. doi: 10.1186/s12864-024-09993-7 (PMC10795378; doi:10.1186/s12864-024-09993-7)
Supplement: Supplementary file 1 — Additional file 1: Figure S1. MAF distribution of Maiwa, Yushu, and Huanhu yaks. Figure S2. The kinship of whole population based on the SNPs. Figure S3. 2 D PCA plot for three yaks population. The A was drawn with PC1 against PC2. The B was drawn with PC1 against PC3. The C was drawn with PC3 against PC2. Figure S4. The percentage between PCs explained variance and total phenotype variance. Figure S5. Enriched network between Gene Ontology terms by P-values. Figure S6. Gene Ontology terms by P-values. Then 0.3 kappa score was applied as the threshold to cast the tree into term clusters. Figure S7. The LD decay of three yaks population with adjust R2 values. Table S1. Detected sweeps and associated gene names. Table S2. GO terms and descriptions. [file 12864_2024_9993_MOESM1_ESM.docx]

Figure S1. MAF distribution of Maiwa, Yushu, and Huanhu yaks.

Figure S2. The kinship of whole population based on the SNPs.


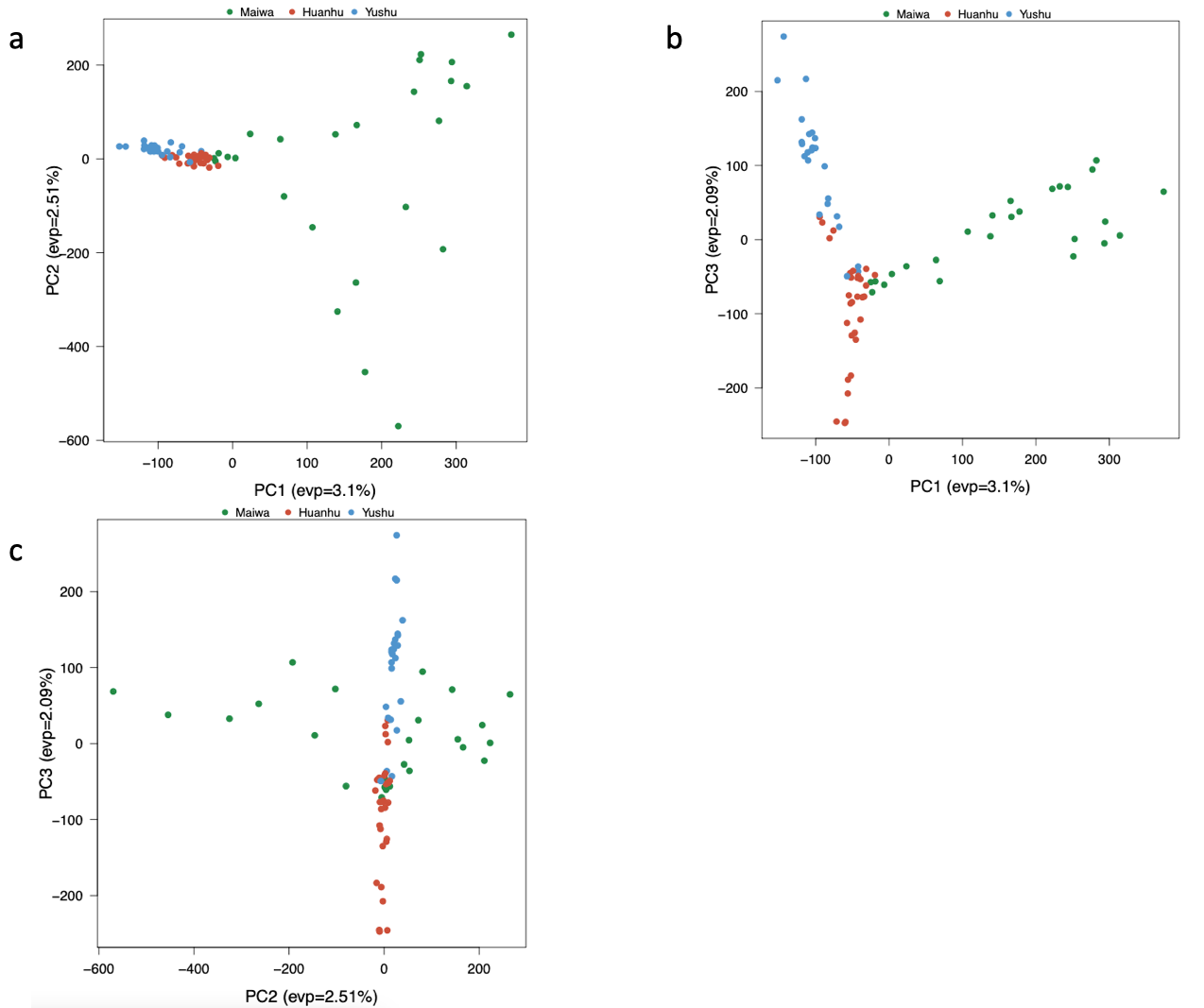


Figure S3. 2 D PCA plot for three yaks population. The A was drawn with PC1 against PC2. The B was drawn with PC1 against PC3. The C was drawn with PC3 against PC2.

Figure S4. The percentage between PCs explained variance and total phenotype variance.


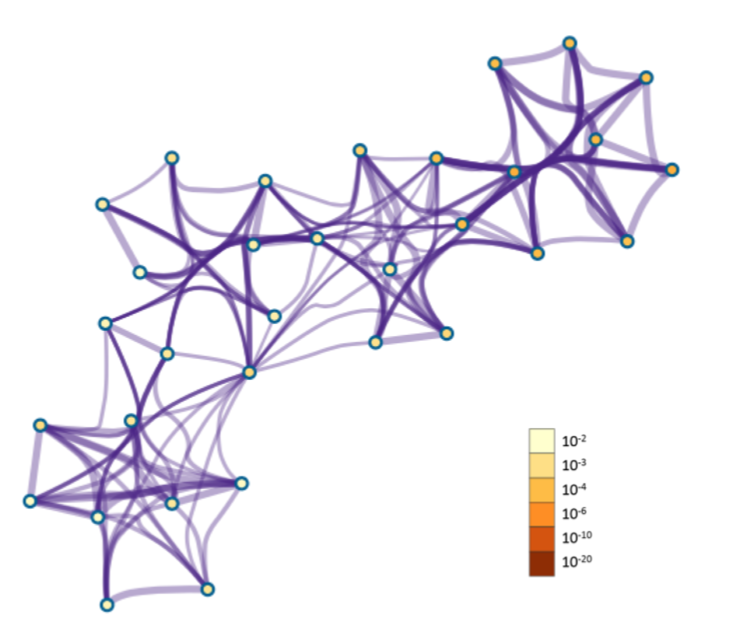


Figure S5. Enriched network between Gene Ontology terms by P-values.


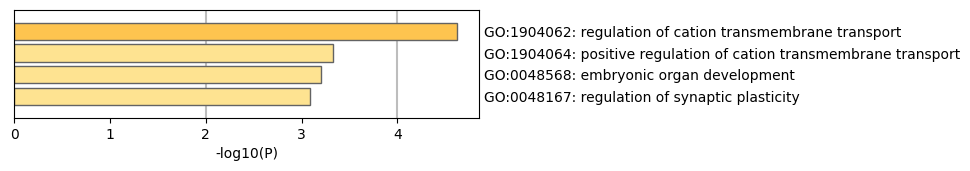


Figure S6. Gene Ontology terms by P-values. Then 0.3 kappa score was applied as the threshold to cast the tree into term clusters.


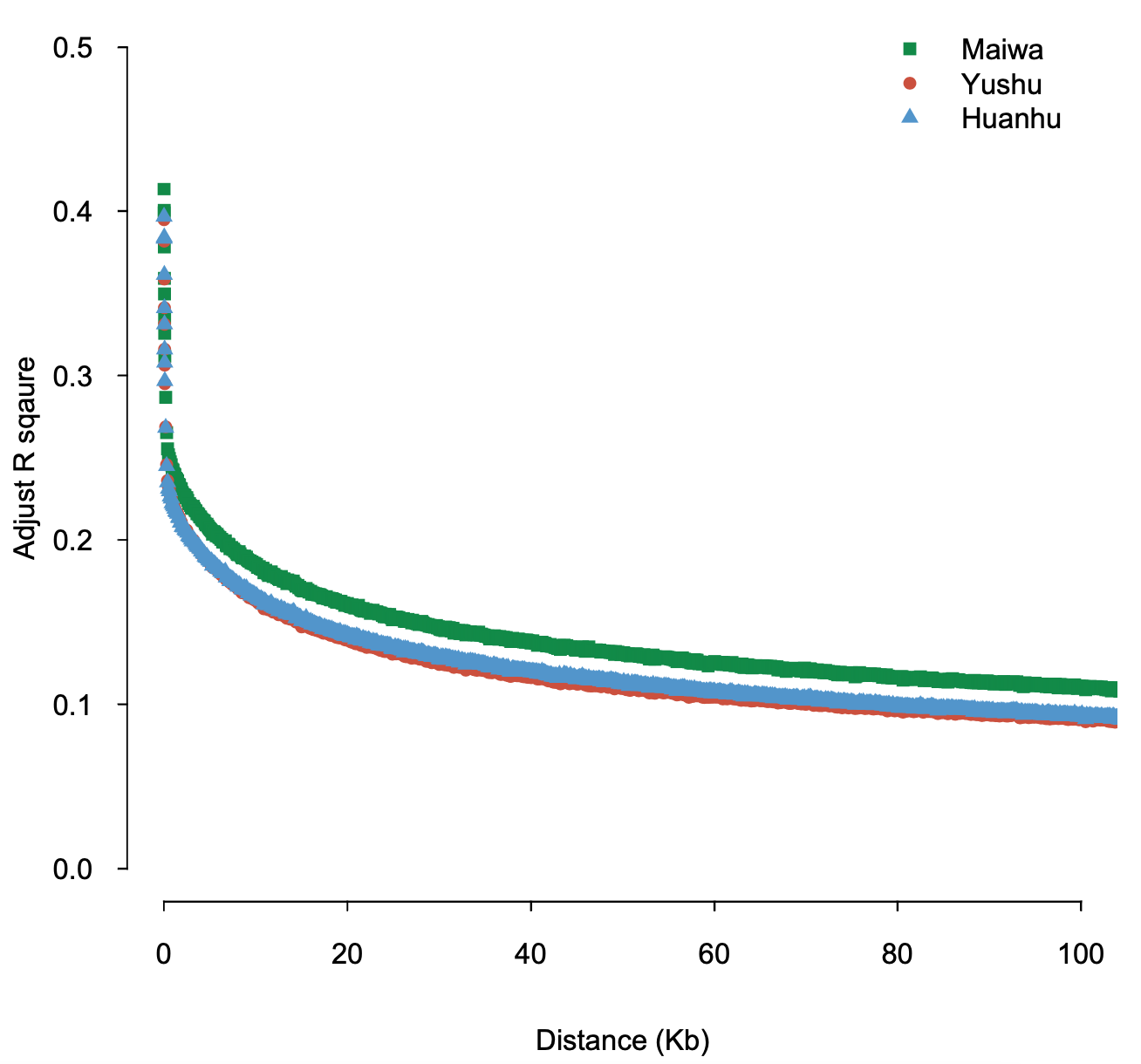


Figure S7. The LD decay of three yaks population with adjust R2 values

Table S1. Detected sweeps and associated gene names

The Chr indicated chromosome number. The Pos indicate sweeps position. The Values indicate separation level from 0, the positive and negative indicate relative separation direction from reference population (Ref) to comparison population (Com). Methods indicate detected methods. G_name indicate gene name with signals. The Type indicate separation type.

| Chr | Pos | Values | Methods | G_name | Type | Ref | Com |
| --- | --- | --- | --- | --- | --- | --- | --- |
| 1 | 26197536 | -0.82 | xpehh | NA | between | Maiwa | Yushu |
| 1 | 26197536 | -0.71 | xpnsl | NA | between | Maiwa | Yushu |
| 1 | 26200117 | -0.80 | xpehh | NA | between | Maiwa | Huanhu |
| 1 | 26222915 | -0.73 | xpnsl | NA | between | Maiwa | Huanhu |
| 1 | 93431892 | -0.75 | xpehh | NA | between | Maiwa | Yushu |
| 1 | 93431892 | -0.72 | xpnsl | NA | between | Maiwa | Yushu |
| 1 | 93977374 | -0.70 | xpehh | NA | between | Maiwa | Huanhu |
| 1 | 117293655 | -0.77 | xpehh | NA | between | Maiwa | Huanhu |
| 1 | 117300010 | -0.71 | xpehh | NA | between | Maiwa | Huanhu |
| 1 | 117512327 | -0.72 | xpehh | NA | between | Maiwa | Huanhu |
| 1 | 166952520 | -0.83 | xpehh | ANKRD28 | between | Maiwa | Yushu |
| 1 | 166988217 | -0.70 | xpnsl | ANKRD28 | between | Maiwa | Yushu |
| 1 | 167076159 | -0.74 | xpehh | ANKRD28 | between | Maiwa | Huanhu |
| 1 | 167076159 | -0.71 | xpnsl | ANKRD28 | between | Maiwa | Huanhu |
| 2 | 6996846 | 0.63 | xpehh | EPHB2 | between | Maiwa | Huanhu |
| 2 | 7014352 | 0.62 | xpnsl | EPHB2 | between | Maiwa | Yushu |
| 2 | 18076291 | -1.68 | ihs | NA | within | NA | NA |
| 2 | 23943662 | 0.55 | xpnsl | NA | between | Maiwa | Huanhu |
| 2 | 35466177 | -0.68 | xpnsl | SMARCAL1 | between | Maiwa | Huanhu |
| 2 | 35466849 | -0.70 | xpehh | SMARCAL1 | between | Maiwa | Huanhu |
| 2 | 38928808 | -1.75 | ihs | NA | within | NA | NA |
| 2 | 56904475 | -1.75 | ihs | HECW2 | within | NA | NA |
| 2 | 60507138 | 1.53 | ihs | NA | within | NA | NA |
| 2 | 79807850 | -0.68 | xpnsl | NCKAP5 | between | Maiwa | Huanhu |
| 2 | 79845191 | -0.70 | xpehh | NCKAP5 | between | Maiwa | Yushu |
| 2 | 114535365 | -0.66 | xpnsl | NA | between | Maiwa | Huanhu |
| 2 | 124648801 | -0.78 | xpehh | NA | between | Maiwa | Huanhu |
| 2 | 124648801 | -0.70 | xpnsl | NA | between | Maiwa | Yushu |
| 2 | 139729919 | -0.65 | xpnsl | ZSWIM2 | between | Maiwa | Huanhu |
| 3 | 5463509 | -1.89 | ihs | CD1E | within | NA | NA |
| 3 | 5528477 | -1.78 | ihs | CD1E | within | NA | NA |
| 3 | 51204751 | 0.62 | xpehh | NA | between | Maiwa | Yushu |
| 3 | 51204751 | 0.57 | xpnsl | NA | between | Maiwa | Huanhu |
| 3 | 72875609 | -0.72 | xpehh | NA | between | Maiwa | Yushu |
| 3 | 123469050 | -0.64 | xpnsl | INPP5D | between | Maiwa | Yushu |
| 4 | 7222596 | -1.78 | ihs | NA | within | NA | NA |
| 4 | 12908337 | -0.73 | xpehh | NA | between | Maiwa | Huanhu |
| 4 | 46398253 | -1.00 | xpehh | HECW1 | between | Maiwa | Yushu |
| 4 | 46398253 | -0.86 | xpnsl | HECW1 | between | Maiwa | Yushu |
| 4 | 46400419 | -0.95 | xpehh | HECW1 | between | Maiwa | Huanhu |
| 4 | 46409672 | -0.85 | xpnsl | HECW1 | between | Maiwa | Yushu |
| 4 | 46528305 | -0.71 | xpnsl | NA | between | Maiwa | Huanhu |
| 4 | 49812274 | 1.52 | ihs | NA | within | NA | NA |
| 4 | 60118767 | -1.89 | ihs | ADCYAP1R1 | within | NA | NA |
| 4 | 71003259 | 1.57 | ihs | NA | within | NA | NA |
| 4 | 76819711 | 1.86 | ihs | NA | within | NA | NA |
| 4 | 86530168 | -1.75 | ihs | NA | within | NA | NA |
| 5 | 33932146 | -1.68 | ihs | BTBD11 | within | NA | NA |
| 5 | 76151301 | 2.88 | pi | NA | within | NA | NA |
| 6 | 49717062 | 0.65 | xpehh | NA | between | Maiwa | Yushu |
| 6 | 52088844 | 0.79 | xpehh | NA | between | Maiwa | Yushu |
| 6 | 52088844 | 0.75 | xpnsl | NA | between | Maiwa | Yushu |
| 6 | 52101262 | 0.57 | xpnsl | NA | between | Maiwa | Yushu |
| 6 | 52205368 | 0.57 | xpnsl | KIT | between | Maiwa | Huanhu |
| 6 | 52205943 | 0.62 | xpehh | KIT | between | Maiwa | Huanhu |
| 6 | 54464344 | -0.69 | xpnsl | NA | between | Maiwa | Huanhu |
| 6 | 94055963 | 2.05 | ihs | GRID2 | within | NA | NA |
| 6 | 103559068 | -0.66 | xpnsl | NA | between | Maiwa | Huanhu |
| 6 | 122290901 | 3.02 | pi | NA | within | NA | NA |
| 6 | 122312901 | 2.86 | pi | NA | within | NA | NA |
| 6 | 122834272 | 0.62 | xpehh | NA | between | Maiwa | Huanhu |
| 7 | 5262501 | 2.28 | ihs | NA | within | NA | NA |
| 7 | 5287901 | 2.89 | pi | NA | within | NA | NA |
| 7 | 5354701 | 3.24 | pi | NA | within | NA | NA |
| 7 | 5371993 | 0.76 | xpehh | NA | between | Maiwa | Huanhu |
| 7 | 5372152 | 0.57 | xpnsl | NA | between | Maiwa | Huanhu |
| 8 | 802021 | 0.63 | xpehh | NA | between | Maiwa | Huanhu |
| 8 | 50486021 | -1.68 | ihs | NA | within | NA | NA |
| 8 | 50648090 | -1.69 | ihs | NA | within | NA | NA |
| 8 | 89797322 | 1.64 | ihs | NA | within | NA | NA |
| 9 | 17088009 | 1.82 | ihs | NA | within | NA | NA |
| 9 | 36621397 | -0.66 | xpnsl | NA | between | Maiwa | Huanhu |
| 9 | 66333150 | 0.57 | xpnsl | NA | between | Maiwa | Huanhu |
| 9 | 83369220 | 1.55 | ihs | NA | within | NA | NA |
| 9 | 91359375 | -0.72 | xpehh | ANTXR1 | between | Maiwa | Huanhu |
| 9 | 111721551 | 1.52 | ihs | NA | within | NA | NA |
| 9 | 112813181 | -1.70 | ihs | NA | within | NA | NA |
| 10 | 17992752 | 1.66 | ihs | NA | within | NA | NA |
| 10 | 39819537 | 0.68 | xpehh | FAXC | between | Maiwa | Yushu |
| 10 | 39825619 | 0.63 | xpnsl | FAXC | between | Maiwa | Huanhu |
| 10 | 43688367 | 0.61 | xpehh | NA | between | Maiwa | Yushu |
| 10 | 43696368 | 0.54 | xpnsl | NA | between | Maiwa | Huanhu |
| 10 | 74078385 | 0.56 | xpnsl | NA | between | Maiwa | Yushu |
| 10 | 74084953 | 0.65 | xpehh | NA | between | Maiwa | Yushu |
| 10 | 91430558 | -0.81 | xpehh | NA | between | Maiwa | Huanhu |
| 10 | 91435377 | -0.69 | xpnsl | NA | between | Maiwa | Huanhu |
| 10 | 95107601 | 3.44 | pi | NA | within | NA | NA |
| 10 | 95134244 | -1.69 | ihs | NA | within | NA | NA |
| 10 | 109903120 | -1.70 | ihs | NA | within | NA | NA |
| 11 | 452850 | 1.74 | ihs | NA | within | NA | NA |
| 11 | 1138969 | -1.73 | ihs | NA | within | NA | NA |
| 11 | 25648401 | 3.56 | pi | NA | within | NA | NA |
| 11 | 27166861 | -0.65 | xpnsl | NA | between | Maiwa | Huanhu |
| 12 | 47886296 | -1.95 | ihs | NA | within | NA | NA |
| 12 | 47964804 | -1.79 | ihs | LAMA5 | within | NA | NA |
| 12 | 48012806 | -1.69 | ihs | OSBPL2 | within | NA | NA |
| 12 | 53318378 | 0.55 | xpnsl | NA | between | Maiwa | Huanhu |
| 12 | 53341498 | 0.61 | xpehh | NA | between | Maiwa | Huanhu |
| 12 | 69510751 | -0.68 | xpnsl | NA | between | Maiwa | Huanhu |
| 13 | 35287673 | -1.76 | ihs | NA | within | NA | NA |
| 13 | 35301146 | -1.74 | ihs | ART1 | within | NA | NA |
| 13 | 78786309 | -1.73 | ihs | NA | within | NA | NA |
| 14 | 81901 | 2.90 | pi | NA | within | NA | NA |
| 14 | 179801 | 2.89 | pi | NA | within | NA | NA |
| 14 | 203955 | 1.79 | ihs | NA | within | NA | NA |
| 14 | 273801 | 3.00 | pi | NA | within | NA | NA |
| 14 | 58503983 | -0.68 | xpnsl | NA | between | Maiwa | Huanhu |
| 14 | 88713801 | 3.27 | pi | NA | within | NA | NA |
| 15 | 1921101 | 1.75 | ihs | NA | within | NA | NA |
| 16 | 200201 | 2.79 | pi | NA | within | NA | NA |
| 16 | 456317 | 0.63 | xpehh | NA | between | Maiwa | Huanhu |
| 16 | 456317 | 0.59 | xpnsl | NA | between | Maiwa | Huanhu |
| 16 | 10446402 | -0.73 | xpehh | ARFIP1 | between | Maiwa | Huanhu |
| 16 | 10459031 | -0.69 | xpnsl | NA | between | Maiwa | Huanhu |
| 16 | 37780578 | 1.06 | xpehh | NA | between | Maiwa | Huanhu |
| 16 | 37792886 | 0.77 | xpnsl | NA | between | Maiwa | Huanhu |
| 16 | 37811265 | 0.78 | xpnsl | SPATA5 | between | Maiwa | Yushu |
| 16 | 37836642 | 0.82 | xpehh | SPATA5 | between | Maiwa | Yushu |
| 16 | 38028224 | 0.56 | xpnsl | SPATA5 | between | Maiwa | Huanhu |
| 16 | 38516482 | 0.70 | xpehh | NA | between | Maiwa | Huanhu |
| 16 | 38516482 | 0.63 | xpnsl | NA | between | Maiwa | Huanhu |
| 16 | 39059983 | 0.68 | xpehh | NA | between | Maiwa | Yushu |
| 16 | 39068888 | 0.60 | xpnsl | NA | between | Maiwa | Yushu |
| 16 | 39194168 | 0.68 | xpehh | NA | between | Maiwa | Yushu |
| 16 | 39194168 | 0.66 | xpnsl | NA | between | Maiwa | Yushu |
| 16 | 39463664 | 0.74 | xpehh | NA | between | Maiwa | Huanhu |
| 16 | 39463664 | 0.62 | xpnsl | NA | between | Maiwa | Huanhu |
| 16 | 39517819 | 0.68 | xpehh | NA | between | Maiwa | Yushu |
| 16 | 39517819 | 0.68 | xpnsl | NA | between | Maiwa | Yushu |
| 16 | 47640550 | -0.69 | xpehh | NA | between | Maiwa | Huanhu |
| 16 | 78456301 | 2.82 | pi | NA | within | NA | NA |
| 17 | 945760 | -1.76 | ihs | NA | within | NA | NA |
| 17 | 47768839 | 1.55 | ihs | NA | within | NA | NA |
| 17 | 76104501 | 2.82 | pi | NA | within | NA | NA |
| 18 | 330373 | 1.16 | xpehh | NA | between | Maiwa | Huanhu |
| 18 | 330373 | 0.62 | xpnsl | NA | between | Maiwa | Huanhu |
| 18 | 636420 | 0.78 | xpehh | NA | between | Yushu | Huanhu |
| 18 | 769901 | 2.77 | pi | NA | within | NA | NA |
| 18 | 830601 | 3.11 | pi | NA | within | NA | NA |
| 18 | 846107 | 0.71 | xpehh | NA | between | Yushu | Huanhu |
| 18 | 54284489 | -0.65 | xpnsl | GRHL2 | between | Maiwa | Huanhu |
| 18 | 54286347 | -0.70 | xpehh | GRHL2 | between | Maiwa | Huanhu |
| 18 | 75722475 | 1.81 | ihs | NA | within | NA | NA |
| 19 | 24269 | 1.55 | ihs | NA | within | NA | NA |
| 19 | 45701 | 3.71 | pi | NA | within | NA | NA |
| 19 | 108613 | -1.74 | ihs | NA | within | NA | NA |
| 19 | 23103987 | -0.70 | xpehh | AOC2 | between | Maiwa | Yushu |
| 19 | 23130356 | -0.67 | xpnsl | PSME3 | between | Maiwa | Yushu |
| 19 | 25006179 | -1.70 | ihs | NA | within | NA | NA |
| 19 | 44570158 | 1.64 | ihs | NA | within | NA | NA |
| 20 | 13557112 | -0.71 | xpehh | NA | between | Maiwa | Yushu |
| 20 | 13584336 | -0.70 | xpnsl | NA | between | Maiwa | Yushu |
| 20 | 13630195 | -0.68 | xpnsl | NA | between | Maiwa | Huanhu |
| 20 | 13632961 | -0.72 | xpehh | NA | between | Maiwa | Huanhu |
| 20 | 16395386 | -1.91 | ihs | NA | within | NA | NA |
| 20 | 34157060 | -0.71 | xpehh | TCF25 | between | Maiwa | Huanhu |
| 20 | 35811244 | -1.74 | ihs | KLHDC4 | within | NA | NA |
| 20 | 52549816 | -1.88 | ihs | RPS9 | within | NA | NA |
| 20 | 52831011 | 1.71 | ihs | NA | within | NA | NA |
| 20 | 67219695 | -0.70 | xpehh | NA | between | Maiwa | Huanhu |
| 21 | 61479109 | -0.77 | xpehh | NA | between | Maiwa | Huanhu |
| 21 | 63075515 | 1.54 | ihs | NA | within | NA | NA |
| 23 | 10896552 | 0.55 | xpnsl | NA | between | Maiwa | Huanhu |
| 23 | 10901624 | 0.56 | xpnsl | NA | between | Maiwa | Huanhu |
| 23 | 52038956 | 0.61 | xpnsl | NA | between | Maiwa | Huanhu |
| 23 | 52039606 | 0.66 | xpehh | NA | between | Maiwa | Huanhu |
| 24 | 6854149 | 1.80 | ihs | NA | within | NA | NA |
| 24 | 6924313 | 1.81 | ihs | VARS2 | within | NA | NA |
| 24 | 7001700 | 1.56 | ihs | NA | within | NA | NA |
| 24 | 8717980 | 1.55 | ihs | NA | within | NA | NA |
| 25 | 1948798 | 0.62 | xpehh | NA | between | Yushu | Huanhu |
| 26 | 18466298 | -0.72 | xpehh | NA | between | Maiwa | Huanhu |
| 26 | 18813071 | -0.66 | xpnsl | NA | between | Maiwa | Huanhu |
| 26 | 44923251 | 1.72 | ihs | NA | within | NA | NA |
| 27 | 114130 | 0.72 | xpnsl | NA | between | Maiwa | Yushu |
| 27 | 114682 | 1.25 | xpehh | NA | between | Maiwa | Yushu |
| 27 | 531958 | -1.70 | ihs | NA | within | NA | NA |
| 27 | 8462874 | -0.67 | xpnsl | RYR2 | between | Yushu | Huanhu |
| 27 | 8587924 | -0.65 | xpnsl | RYR2 | between | Maiwa | Yushu |
| 27 | 8595400 | -0.70 | xpehh | RYR2 | between | Maiwa | Yushu |
| 28 | 86471 | 1.57 | ihs | NA | within | NA | NA |
| 28 | 120890 | 0.67 | xpehh | NA | between | Maiwa | Yushu |
| 28 | 124761 | 0.64 | xpnsl | NA | between | Maiwa | Yushu |
| 28 | 128201 | 2.93 | pi | NA | within | NA | NA |
| 28 | 12645270 | 0.55 | xpnsl | NA | between | Maiwa | Yushu |
| 28 | 12679216 | 0.63 | xpehh | NA | between | Maiwa | Yushu |
| 30 | 4429501 | 3.13 | pi | NA | within | NA | NA |
| 30 | 4535280 | 0.91 | xpehh | NA | between | Maiwa | Yushu |
| 30 | 4535280 | 0.59 | xpnsl | NA | between | Maiwa | Yushu |
| 30 | 4742683 | -1.70 | ihs | NA | within | NA | NA |
| 30 | 19059409 | 1.87 | ihs | NA | within | NA | NA |
| 30 | 19076601 | 2.75 | pi | NA | within | NA | NA |
| 30 | 27159279 | 1.75 | ihs | NA | within | NA | NA |
| 30 | 55777501 | 2.91 | pi | NA | within | NA | NA |
| 30 | 55804001 | 3.02 | pi | NA | within | NA | NA |
| 30 | 55858309 | 1.75 | ihs | NA | within | NA | NA |
| 30 | 97730501 | 3.89 | pi | NA | within | NA | NA |
| 30 | 97962201 | 3.43 | pi | NA | within | NA | NA |
| 30 | 98055301 | 3.08 | pi | NA | within | NA | NA |
| 30 | 123590901 | 2.91 | pi | NA | within | NA | NA |
| 30 | 123604301 | 3.13 | pi | NA | within | NA | NA |
| 30 | 130133101 | 2.99 | pi | NA | within | NA | NA |
| 30 | 135761401 | 3.02 | pi | NA | within | NA | NA |

Table S2 GO terms and descriptions

| GO | Category | Description | Count | % | Log10(P) | Log10(q) |
| --- | --- | --- | --- | --- | --- | --- |
| GO:1904062 | GO Biological Processes | regulation of cation transmembrane transport | 5 | 18.52 | -4.63 | -0.5 |
| GO:1904064 | GO Biological Processes | positive regulation of cation transmembrane transport | 3 | 11.11 | -3.32 | -0.23 |
| GO:0048568 | GO Biological Processes | embryonic organ development | 4 | 14.81 | -3.2 | -0.19 |
| GO:0048167 | GO Biological Processes | regulation of synaptic plasticity | 3 | 11.11 | -3.08 | -0.12 |
